# Exploring Motivating Factors for Pursuing Radiation Oncology: A Comparative Analysis of Medical Students and Residents

**DOI:** 10.7759/cureus.67399

**Published:** 2024-08-21

**Authors:** Brandon S Chai, Maryam Dosani, Timothy Kong, Paris-Ann Ingledew

**Affiliations:** 1 Surgery, University of British Columbia, Vancouver, CAN; 2 Radiation Oncology, BC Cancer, Victoria Centre, Victoria, CAN; 3 Radiation Oncology, BC Cancer, Vancouver Centre, Vancouver, CAN

**Keywords:** residents, medical students, mentorship, professional identity, specialty selection, medical education, radiation oncology

## Abstract

Purpose

Increasing medical student (MS) interest in radiation oncology (RO) is important to meet the rising demand for radiation oncologists. Understanding the factors that drive MS to pursue RO is crucial. This study compares motivating factors between MS and RO residents to inform interventions to increase recruitment and sustained interest in the specialty.

Methods

Data from two similar studies investigating factors motivating MS and residents to pursue RO were analyzed. The first study surveyed Canadian RO residents to characterize enablers when applying for RO residency. The second study analyzed application essays from MS applying to an RO studentship. A mixed methods approach was used to compare themes (“career aspects,” “prior exposure,” and “personal experiences”) between the datasets.

Results

Qualitative analysis demonstrated that both MS and residents identified “career aspects” as the most common theme facilitating interest in RO careers. “Multidisciplinary work” and “direct clinical contact and patient care” were prominent sub-themes. MS emphasized “serious illness and palliative care” and “advanced technology,” while residents prioritized RO as a “rewarding career.” “Prior exposure,” particularly through shadowing/observerships, was more important for MS than residents who valued clinical experiences. Practical career considerations including “mentorship” and “career satisfaction and lifestyle” were significant motivators for residents.

Conclusion

MS value content-based aspects of RO and emphasize shadowing. In contrast, RO residents prioritize lifestyle-based considerations. These differences highlight the opportunity for intervention throughout medical training to sustain interest in RO and facilitate applications to RO residency programs.

## Introduction

Radiotherapy is essential to comprehensive cancer care and is indicated in over 50% of cancer cases. Indications for radiotherapy continue to increase [1,2]. As such, there is an increasing demand for radiation oncologists to provide quality cancer care. In fact, Loewen et al. (2023) suggested that the demand for radiation oncologists will outgrow the supply in Canada, even with the current staffing expansion efforts [3]. 

In order to train enough radiation oncologists to meet this demand, it is necessary to recruit medical students (MS) for the specialty. However, there have been fluctuations in MS applications to radiation oncology (RO) in both Canada and the United States, resulting in unfilled positions in the specialty [4-6]. Though the number of unfilled positions has improved in recent years, it remains important to direct efforts toward increasing MS interest in RO and supporting recruitment strategies [7].

Investigating the factors that drive MS to pursue a career in RO can inform interventions to encourage future applicants. Kong et al. (2020) qualitatively evaluated the essays from junior MS applying to the Canadian Associate of Radiation Oncology - Canadian Radiation Oncology Foundation (CARO-CROF) Pamela Catton studentship from the years 2014-2019 to explore motivating factors for pursuing RO [8]. Similarly, Dosani et al. (2020) administered a mixed methods survey to Canadian RO residents from the 2015-2019 match years to characterize “enabling” factors to apply to RO, from the perspective of current RO residents [9]. Both studies provide valuable insights into the factors that motivate MS to pursue RO from different stages in training. Thus, a comparison of the factors between both samples would provide further insight into how motivating factors change throughout training and where to best allocate efforts to maximize interest in RO.

The current study is a secondary data analysis of the data presented by Kong et al. (2020) and Dosani et al. (2020) and aims to compare the factors that motivated MS to pursue RO in comparison to residents [8,9]. Comparison of these findings will identify enabling factors that remain important through medical training and provide insight into how these factors change throughout medical training. As such, these findings will inform resource allocation for recruitment strategies to RO residency programs and identify opportunities to increase MS interest and support RO residents through their training.

## Materials and methods

Study design

This secondary data analysis obtained data from studies conducted by Kong et al. (2020) and Dosani et al. (2020) [8,9]. Dosani et al. (2020) conducted a mixed methods survey, which was administered to Canadian RO residents (2015-2019 match) via Program Directors to characterize RO enablers when applying to RO residency [9]. The survey was adapted from the expanded conceptual framework of MS’ primary care career choice for relevance to RO [10]. The survey was hosted on Qualtrics and included 58 questions, primarily Likert-type multiple choice questions using a scale of 1 to 5 (1 = not important, 5 = very important) with one optional free text response, which asked: “Did any other factors influence your decision to pursue radiation oncology?” Kong et al. (2020) obtained MS application essays from the CARO Education Committee for the CARO-CROF Dr. Pamela Catton studentship [8]. Essays were obtained from application years 2014-2019. The students provided a short essay (maximum 500 words) responding to the prompt: “What interests them in pursuing this RO studentship.” Institutional Research Ethics Board (REB) approval was obtained from the University of British Columbia for both studies.

Quantitative data from Dosani et al. (2020) was analyzed using descriptive statistics [9]. Free text responses in both data sets were evaluated using a grounded theory approach to qualitative analysis [11]. Themes and sub-themes were established via a systematic approach using multiple independent reviewers [12]. Dosani et al. (2020) used two staff radiation oncologists who independently reviewed all free-text responses [9]. Kong et al. (2020) used three staff radiation oncologists for independent review, two of which reviewed a random subset of 10 essays while the other reviewed all essays [8]. In both studies, discrepancies in coding were resolved through discussion among reviewers to achieve consensus, followed by re-evaluation of the responses to ensure consistency [8,9].

Data analysis

The current study used a mixed methods approach to compare the importance of themes and sub-themes presented in each study. First, the results of each study were compared to identify common and discrepant themes. Then, the sources of data that informed these themes were evaluated from each study. The data sources by Dosani et al. (2020) were primarily quantitative using Likert-type items on a scale from 1 to 5 (1 = not important and 5 = very important) with supplemental qualitative data from free-text responses [9]. The results from Kong et al. (2020) were only qualitative and were derived from the MS application essays [8]. The importance of each quantitative item in Dosani et al. (2020) was then determined using the means of each item (4-5 = high importance; 3-3.9 = moderate importance; 2-2.9 = low importance; 1-1.9 = very low importance) [9]. The importance of the theme presented in qualitative data was determined using the frequency of each theme in the free-text responses of Dosani et al. (2020) and essays by Kong et al. (2020) [8,9]. Quantitative items with higher mean scores and qualitative themes with a higher frequency were deemed more important. Finally, the importance of each theme was grossly compared between each study to identify themes that 1) were important for both MS and RO residents, 2) were important for MS and not RO residents, and 3) were important for RO residents but not MS.

## Results

Dosani et al. (2020) received 65 of 123 (52.8% response rate) responses from Canadian RO residents [9]. Most (52.2%) of these students were in their first or second year of RO residency. Thirty-three of the 65 (50.7%) responses completed the free text question. Kong et al. (2020) obtained 66 essays for analysis, with 81% of the responses from second-year MS and 19% from first-year MS [8]. The demographics from each study are described (Table 1).

**Table 1:** Demographics of respondents in Kong et al. (2020) and Dosani et al. (2020)

		Number of respondents (percent of total)	
		Dosani et al. (2020) [9]	Kong et al. (2020) [8]
Year of training	MS 1	-	13 (19.7)
	MS 2	-	53 (80.3)
	MS 3	-	0 (0)
	MS 4	-	0 (0)
	PGY 1-2	34 (52.2)	-
	PGY 3	10 (13.4)	-
	PGY 4	13 (19.4)	-
	PGY 5	10 (14.9)	-
Age (years old)	20-24	6 (8.8)	Not available
	25-29	46 (67.7)	Not available
	30-34	11 (16.2)	Not available
	35-39	3 (4.4)	Not available
	≥40	2 (2.9)	Not available
Gender	Female	24 (35.8)	39 (59.1)
	Male	42 (62.3)	27 (40.9)
	Self-described	1 (1.5)	0 (0)

Comparison of major themes

 Both studies followed a similar distribution regarding the frequency and importance of major themes (“career aspects,” “prior exposure,” and “personal experiences") described by Kong et al. (2020) [8,9]. “Career aspects” was the most common and important major theme from MS and RO residents, appearing in 82% of MS essays and discussed by 45% of RO residents with high mean importance of 3.7 from the corresponding 5-point Likert items. The theme “prior exposure” was the second most common theme, identified in 74% of MS essays and mentioned in 12% of RO resident responses with a mean importance of 3.0. Finally, “personal experiences” was the least common theme, found in only 26% of MS essays and in 3% of RO resident responses. There were no Likert-type items relevant to personal experiences with oncology in this survey.

Comparison of sub-themes

Career Aspects

“Multidisciplinary work” and “direct clinical contact and patient care” were the two most common “career aspect” sub-themes exhibited by MS from Kong et al. (2020), mirroring findings from RO residents from Dosani et al. (2020) [8,9]. The “rewarding career” sub-theme was a very important sub-theme for RO residents with high mean importance of 4.2 from the corresponding 5-point Likert item but was less frequently highlighted by MS. The “serious illness and palliative care” and “advanced technology” sub-themes were important to MS as per Kong et al. (2020); however, these were only mentioned in one (3%) and two (6%) of RO resident free text with no corresponding quantitative data [8].

Prior Exposure

The “shadowing/observerships” sub-theme was very important for MS as per Kong et al. (2020); however, it was not mentioned in the free text responses from RO residents and had a low score of 2.9 on the corresponding Likert item [8]. Similarly, “oncology-specific research” was a frequent sub-theme for MS but had low importance for RO residents with a mean Likert score of 2.6. On the contrary, “relevant clinical experiences” were less important for MS compared to RO residents who deemed this sub-theme more important with a moderate Likert score of 3.5. “Under-exposure” of RO in the medical school curriculum was a greater factor for MS to pursue RO than RO residents as it was only mentioned once by RO residents and had a low importance score of 2.0.

Personal Experiences

Personal experience sub-themes such as an individual’s “own diagnosis,” “family,” and “community involvement” were more frequently identified as factors for MS than for RO residents. There were no corresponding Likert items in the data set from Dosani et al. (2020) and there was only one free-text response that mentioned an experience in oncology from a family member [9].

Radiation oncology resident factors: practical career considerations

RO residents in Dosani et al. (2020) highlighted themes that were not illustrated in the MS essays from Kong et al. (2020) [8,9]. The additional major theme “practical career considerations” appeared in 82% of the free-text responses from RO residents [9]. The most frequent sub-theme was “mentorship” (from staff radiation oncologists or RO residents), which was present in 33% of all free-text responses and had a moderate importance score of 3.9. “Career satisfaction and lifestyle” was the second most common sub-theme, appearing in 30% of free-text responses and an importance score of 3.4. “Career flexibility and variability” (18%) and “practical factors” (15%; e.g., the job market, direct entry specialty, and specialty) were relatively less common in the free-text responses than the other practical career considerations but more common than any sub-theme from prior exposure and personal experiences. Table 2 provides an overview of the main themes and sub-themes present in each study.

**Table 2:** Summary of themes and sub-themes valued by MS (Dosani et al., 2020) and RO residents (Kong et al., 2020) ^a^Denotes most important themes ^b^Denotes most important sub-themes MS, medical students; RO, radiation oncology

	Sub-themes	
Theme	MS [9]	RO residents [8]
Career aspects^a^	Multidisciplinary work^b^	Multidisciplinary work
	Direct patient care^b^	Direct patient care
	Serious illness/palliative care	Rewarding career^b^
	Advanced technology	
Prior exposure	Shadowing/observership^b^	Relevant clinical experiences^b^
	Oncology-specific research	
	Under-exposure	
Personal experiences	Own diagnosis	Not applicable
	Family	
	Community involvement	
Practical career considerations^a^	Not Applicable	Mentorship^b^
		Satisfaction and lifestyle^b^
		Flexibility and variability
		Practical factors

## Discussion

This secondary data analysis compared motivating factors for MS and residents to pursue RO [8,9]. Comparing these enablers sheds light on how factors evolve throughout training. The expanded conceptual framework developed by Pfarrwaller et al. (2017) for MS’ primary care career choice is one of the first models to include the dimension of time and longitudinal evolution throughout training [10]. Although the framework was intended to characterize decision-making for choosing a career in primary care, the findings in our study support this model and its generalizability to choosing a career in a medical specialty such as RO.

“Career aspects” was the most common major theme shared between RO residents and MS applying to RO, with “multidisciplinary work” and “direct clinical and patient care” as the most common sub-themes [8,9]. While MS valued advanced technology and serious illness/palliative care, the perception of RO as a rewarding specialty was relatively more important for RO residents than MS [8,9]. Furthermore, the most important enabling factors for RO residents were “practical career considerations” including “mentorship,” “career satisfaction and lifestyle,” “career flexibility and variability,” and “practical factors” [9]. “Mentorship” was a particularly important factor for RO residents and has been previously described as a significant determinant of specialty selection [13-17]. Mentorship can impact specialty selection in a variety of ways including the formation of students’ professional identity, induction of moral elevation (positive emotions experienced when witnessing exceptional role model behavior), and ultimately can motivate students to pursue specialties that they otherwise would not have considered [13,18,19]. Furthermore, the importance of lifestyle factors supports insights from Pfarwaller et al. (2023) who found that priorities evolved from content-based considerations (i.e., what students want to be) to lifestyle-based considerations (i.e., how students want to work) as students proceed through training and develop their professional identity [20]. The emphasis on lifestyle values is reflected by other recent studies, such as a scoping review by Sarikhani et al. (2021) who found that having a controllable lifestyle was one of the most common determinants of specialty selection and was reported in a large proportion (69%) of studies [21-23]. In the context of this study, content-based considerations such as technology and palliative care may help attract junior MS to the specialty; however, lifestyle-based considerations such as perceived career fulfillment, flexibility, and practical factors are more important for senior MS and sustaining interest among residents. Notably, the data from Kong et al. (2020) only included first- and second-year MS, which may explain their emphasis on content-based considerations [8]. Although not traditionally taught in medical school, it is essential for MS to learn about these lifestyle-based factors to help generate interest. This calls to action for RO residents and staff to provide this education, which can take place through extra-curricular settings such as mentorship and may be particularly important during the later years of medical school.

“Prior exposures” in RO were a common theme for both MS and RO residents [8,9]. “Shadowing/observerships” was deemed more important to MS interested in RO than RO residents. On the contrary, current RO residents appraised relevant clinical exposures (i.e., rotations during clerkship) to be much more important than shadowing [9]. This reflects findings from Pfarwaller et al. (2023) who emphasize the importance of gaining practical experience for developing students’ vague image of a specialty to a concrete and realistic understanding [20]. Furthermore, early clinical exposure in medical school has frequently been reported to have positive impacts on MS learning, motivation, and interest in medical specialties [24-26]. This study supports previous findings that practical experiences such as shadowing or clinical rotations play a critical role in developing a student’s professional identity and matching it to a specialty of choice. This highlights the importance of encouraging junior MS to shadow and senior MS to choose RO electives during clerkship. It also provides support to increase the number of RO elective spots, especially those that appear earlier in the clerkship rotation schedule as earlier exposure may impart a greater impact on career selection [27].

Other “prior exposures” such as “oncology-specific research” and “personal experiences” (i.e., “Own diagnoses,” “family,” and “community involvement”) were less important for RO residents but more significant for MS [8,9]. MS have less experience in medicine compared to residents, so their decisions may be influenced more by personal experiences outside of their training. However, these personal experiences may inspire purposeful career exploration in early medical training. This aligns with Cuesta-Briand et al. (2020) who proposed two main types of career decision-making: “planners,” who are more decided on their careers and proactively undertake deliberate actions to achieve career goals, and “explorers” who are more undecided, demonstrate a mix of active and passive behaviors and have a more laidback approach to career decision making [28]. Perhaps prior exposures and personal experiences foster “planner” characteristics in early MS and provide the foundation for subsequent purposeful career exploration, which becomes more significant in the later stages of training.

This study has limitations. Data collection methods differed between studies; Dosani et al. (2020) administered a survey to RO residents, which collected both quantitative and qualitative data, whereas Kong et al. (2020) obtained only qualitative data from MS essays [8,9]. Variations in questions may have introduced bias, making it more difficult to draw conclusions from this comparison. The data was also obtained in different environments, which may have impacted the responses; for example, RO residents may have been more candid with their responses, whereas MS applying to the RO studentship may have been more selective with their responses. Comparing the mixed-method data from Dosani et al. (2020) with the qualitative data from Kong et al. (2020) was challenging given the inherent differences in data characteristics; however, this approach yielded a more comprehensive and quantifiable understanding of motivating factors in comparison to a qualitative or quantitative approach only [8,9]. Finally, both data sets provided cross-sectional instead of longitudinal data, which additionally hinders the assessment of changes over time [8,9]. Given these limitations, the results of this study should be interpreted with caution, suggesting a need for future research using more robust methodologies such as a longitudinal mixed-methods survey.

## Conclusions

This study compares key enabling factors between MS and RO residents and supports pre-existing conceptual frameworks characterizing dynamic MS career decision-making over time. Content-based considerations appeared to be the primary motivating factors for MS pursuing RO, whereas lifestyle-based considerations were more important for RO residents. These differences highlight the opportunity for intervention at various stages of medical training to inform career decision-making, facilitate applications to RO residency programs, and generate sustained interest in RO for residents transitioning to staff positions.
